# Adherence to the Mediterranean diet and psychological wellbeing before and during the COVID-19 pandemic: a prospective analysis of the English Longitudinal Study of Ageing

**DOI:** 10.1136/bmjopen-2025-109599

**Published:** 2026-06-16

**Authors:** Andrew Steptoe, Alanna Jo Shand, Camille Lassale

**Affiliations:** 1 Behavioural Science and Health, University College London, London, UK; 2 Psychology, Institute of Psychiatry, London, England, UK; 3 Environmental Health Over the Lifecourse Programme, Barcelona Institute for Global Health, Barcelona, Spain

**Keywords:** NUTRITION & DIETETICS, Longitudinal studies, MENTAL HEALTH, COVID-19

## Abstract

**Abstract:**

**Objectives:**

Following a Mediterranean-like diet has been associated with lower depression levels but there has been limited study of links with positive states of psychological well-being. We tested whether adherence to a Mediterranean diet was cross-sectionally associated with positive well-being among older adults in England in 2018/2019 and prospectively through the COVID-19 pandemic.

**Design:**

Prospective observational cohort analysis.

**Setting:**

Population study of older people ≥50 years in England.

**Participants:**

3296 participants (46% men) in the English Longitudinal Study of Ageing (ELSA) aged 68.18±7.43 years, who completed a dietary module in 2018/2019 for the assessment of the Mediterranean diet (relative Mediterranean Diet Index (rMED)).

**Primary and secondary outcome measures:**

Psychological well-being measured using the CASP12 at baseline (2018/2019) and again in the early months of the COVID-19 pandemic (2020).

**Results:**

Cross-sectionally, higher rMED scores were associated with greater psychological well-being (β=0.054, p<0.001), independent of depressive symptoms, income, education, physical activity, smoking, self-rated health and limiting long-standing illness. Longitudinal analyses revealed that higher adherence to the Mediterranean diet predicted smaller declines in well-being during the early months of the COVID-19 pandemic (β=0.026, p=0.042), even after accounting for baseline well-being and COVID-19 infection experience.

**Conclusions:**

Greater adherence to a Mediterranean diet was positively associated with psychological well-being among older adults in England. This relationship persisted even during a period of widespread societal stress. Promoting Mediterranean-style dietary patterns may support not only physical but also mental well-being in later life, underscoring the importance of nutritional public health strategies for mental health resilience in ageing populations.

**STRENGTHS AND LIMITATIONS OF THIS STUDY**

- The study used data from a large representative cohort sample of older men and women.
- The availability of multiple sociodemographic, health and behavioural covariates and the longitudinal study design strengthen the conclusions that can be drawn.
- Adjustment for depressive symptoms indicates that the association of Mediterranean diet with psychological well-being encompasses other dimensions of well-being beyond avoidance of distress.
- Dietary data were collected over 2 days and psychological well-being on two occasions but only one during the early months of the COVID-19 pandemic, and these may not be representative.
- Only a subsample of English Longitudinal Study of Ageing participants carried out the dietary assessment, limiting generalisability.

## Introduction

 There is widespread public health and clinical interest in understanding the relevance of nutrition to mental health problems such as depression.^1^ In addition to analyses of specific nutrients such as B vitamins and n-3 polyunsaturated fatty acids or food groups such as ultra-processed foods or fibre-rich foods, much research has focused on composite dietary indices including the Mediterranean diet. There is robust evidence for the protective effect of the Mediterranean diet for primary prevention of depression based on large-scale cross-sectional and longitudinal observational studies.^2 3^ These associations are supported by the reductions in depressive symptoms observed in randomised controlled dietary intervention studies in patients with depression lasting up to 6 months.^4 5^

There is growing understanding that positive psychological well-being is related to future health outcomes independently of negative emotional states such as depression and anxiety.^6^ Positive well-being has been associated with reduced risk of cardiovascular disease and disability and with longer survival among middle-aged and older people, after controlling for sociodemographic factors, physical health and depression or distress.^7 8^ Exploration of the dietary correlates of positive psychological well-being has been limited to date, particularly in older adults, and has been largely confined to simple measures of fruit and vegetable consumption.^9 10^ The first aim of our study was therefore to assess the association between adherence to a Mediterranean diet and psychological well-being in a large population sample of men and women aged 50 years and older in England and to test whether the relationship is independent of depression.

Cross-sectional studies cannot establish the temporal relationship between diet and psychological well-being. Longitudinal analysis may also be inadequate if there is little change in well-being over time. However, the COVID-19 pandemic provided the opportunity to test prospective associations in a more nuanced way. The early months of the pandemic led to an overall deterioration in positive psychological well-being in the UK population, fuelled by fear of illness and by the physical and social restrictions put in place to reduce infection risk.^11 12^ Among older people in England, depression levels increased and positive psychological well-being worsened in the summer of 2020, although with wide variations, and most of these measures reverted to previous levels after the pandemic was over.^13 14^ Various other factors such as systemic inflammation in the years before the pandemic and childhood adversity have been associated with heightened distress during the pandemic,^15 16^ while pre-pandemic resilience appears to have been protective.^17^ We therefore hypothesised that if the Mediterranean diet is related to enhanced positive well-being, then it should be associated with less deterioration in well-being during the early months of the COVID-19 pandemic, and testing this association was our second main objective.

## Method

### Study sample

The English Longitudinal Study of Ageing (ELSA) is a population survey of a representative sample of men and women ranging in age from 50 years to over 90 years, living in the community in England.^18^ Data are collected at 2-year intervals, with new respondents being periodically added to maintain the full age range. Data are primarily collected by computer-assisted personal interviews carried out in participants’ homes supplemented with a self-completion questionnaire, with additional modules on specific issues. ELSA includes three categories of participants: the core nationally representative sample, other individuals in the household who are aged 50 years and older but are not part of the core sample and younger partners. 8557 individuals aged ≥50 years participated in wave 9 (2018/2019), of whom 4254 completed an online nutritional assessment. Online supplemental table 1 provides a comparison between individuals who did and did not complete the nutrition assessment. There was no difference in age between the groups, but people who provided nutrition data were more likely to be men or of white ethnicity, had completed an average 0.58 more years of education and had higher incomes than those who did not provide data. They also had a better self-rated health, were more physically active and less likely to be smokers. Psychological well-being was greater on average and fewer depressive symptoms were reported by participants in the nutritional survey.

Of the individuals who provided data on total energy intake and Mediterranean diet, 67 were excluded because energy intakes were implausibly high or low, applying thresholds recommended in UK Biobank of <800 kcal/day or >4200 kcal/day for men and <600 kcal/day or >3500 kcal/day for women.^19^ Measures of psychological well-being and all covariates bar income were completed by 3953 of the remaining participants. Income in ELSA is only assessed in the core sample, reducing the sample size for cross-sectional analyses to 3296. The primary analyses were carried out on the core sample, but the analyses of the full sample (3,953) are included in online supplemental material.

Data were also collected during the early months of the COVID-19 pandemic (June/July 2020) with an Internet survey that had a response rate of 75%.^13^ Of the participants with a complete nutrition assessment and covariates at wave 9, measures of psychological well-being during the pandemic were available from 3023 individuals (91.6%). Patients and the public were not involved in the design or conduct of this research.

### Dietary assessment

Diet was assessed on two non-consecutive days using the Oxford WebQ, an online 24 hours recall tool used in other large-scale studies in the UK such as UK Biobank and the Million Women Study.^20^ The module contains more than 200 items grouped into 21 food and beverage categories, and respondents report what they have consumed and in what quantities over the previous day from midnight to midnight. Images of portion sizes are included to improve accuracy of reporting. These assessments have been validated against nutritional biomarkers and are acceptable to older respondents.^21 22^ The intake of 21 separate nutrients, including total energy intake (kcal), macronutrients and micronutrients, is computed by multiplying amounts consumed by nutrient contents as specified in standard UK food composition tables.^19 23^ The 2 days of assessment were averaged for these analyses.

Consumption of a Mediterranean diet was quantified using the relative Mediterranean Diet Index (rMED).^24^ This index includes nine components: fruit, vegetables, legumes, cereals, fish, olive oil, meat, dairy and alcohol. Intake of these food groups is expressed as energy density (grams/1000 kcal) and individuals are grouped in tertiles of intake of each food group. For fruit, vegetables, legumes, cereals and fish, a score of 0, 1 or 2 is attributed to participants in the first, second or third tertile, respectively. For meat and dairy, the scoring is reversed. For alcohol, participants falling in the recommended range of ‘moderate drinking’ (range: 5–25 g/day ethanol for women and 10–50 g/day for men) were attributed a score of 2, 0 otherwise. The scoring for olive oil was modified because of the relatively large number of non-consumers. Therefore, 0 was assigned to non-consumers, 1 for subjects below the median (calculated only within olive oil consumers) and 2 for subjects equal to or above this median. Scores ranged from 0 to 18, with higher scores indicating greater adherence to a Mediterranean diet. This index has been widely used and compares favourably with other measures of Mediterranean diets.^25^

### Psychological well-being

Psychological well-being was assessed using the CASP-12, a shortened version of the CASP-19 measure developed for ELSA.^26 27^ This assesses multiple domains of hedonic and eudemonic well-being with items such as ‘I feel that my life has meaning’ and ‘I enjoy the things that I do’. The scale has been widely used and is psychometrically robust.^28 29^ Scores can range from 0 to 36, with higher scores indicating more positive well-being.

### Other measures

Age at the time of wave 9 (2018/19) examination was modelled as a continuous variable in these analyses. Sex was divided into male and female, and ethnicity into white and non-white. Education was assessed as the age at which the participants completed their schooling. Income was measured through a detailed module on multiple sources of income (employment, pensions, investments, welfare benefits, etc) as detailed elsewhere and was analysed by decile.^30^ Participants were asked whether they had any long-standing illness, disability or infirmity and whether this limited their activities in any way; the two items were combined to indicate the presence of a limiting long-standing illness. Subjective health was rated on a five-category scale from excellent to poor, and low self-rated health was defined as a rating of fair or poor. Respondents were asked if they were current smokers, and physical activity was a binary variable defined as carrying out moderate or vigorous activity at least once per week. Depressive symptoms were assessed using the eight-item version of the Center for Epidemiologic Studies Depression scale (CES-D), and scores could range from 0 to 8.^31^ The experience of COVID-19 infection during the early stages of the pandemic could not be assessed objectively because of the absence of antibody testing in that period and so was based on symptom reports, as detailed previously.^32^

### Statistical analysis

The study sample was divided into tertiles of rMED scores (allowing for ties) for descriptive purposes, but continuous scores were used in the main analyses. Differences between rMED groups were analysed by analysis of variance for continuous and χ^2^ tests for categorical variables, respectively. The cross-sectional association of Mediterranean diet and psychological well-being was analysed using multivariable linear regression, with results presented as standardised regression coefficients (β) with standard errors and p values. Three regression models were tested, with CASP-12 scores as the continuous outcome. Model 1 included age, sex, ethnicity and total energy intake (kcal/day) along with the main exposure and the rMED score (also continuous). Model 2 additionally adjusted for demographic, health and behaviour variables, namely income, education, limiting long-standing illness, low self-rated health, smoking and physical activity. Model 3 tested whether associations of diet quality and positive well-being were present after negative mood states were taken into account, so the CES-D was added to the analysis. Missing data on covariates was <0.01%, so a complete case analysis was carried out.

The longitudinal relationship between Mediterranean diet in 2018/2019 and psychological well-being during the COVID-19 pandemic was analysed by using CASP-12 measures taken in June/July 2020 as the outcome variable. In addition to the variables included in the cross-sectional analyses, model 1 included baseline CASP-12 scores and the experience of COVID-19 infection. Models 2 and 3 added the same variables as before.

Supplementary analyses tested the cross-sectional and longitudinal associations in the full sample, after excluding income from the models. We also evaluated whether the longitudinal relationship remained robust if CES-D depression scores obtained during the pandemic were substituted for baseline depression.

## Results

The participants in this analysis were aged 68.18 years on average in 2018/2019 and included slightly more women (53.9%) than men (table 1). The majority of respondents were of white ethnic origin. The age of completing education averaged 16.89 years, and weekly income deciles ranged from £108 to £1306. Over a quarter of respondents (29.3%) reported having a limiting long-standing illness, and 17.9% indicated that they were in fair or poor health. The proportion of current smokers was small (6.3%), and the majority stated that they were physically active. Mediterranean diet scores ranged from 0 to 14. As table 1 indicates, participants with higher rMED scores were older, more educated and had higher household incomes than the lower rMED individuals and were less likely to have limiting long-standing illness and fair or poor self-rated health. Higher rMED scores were related to greater physical activity, lower energy intake and less smoking. There were no differences in gender distribution or the experience of COVID-19 infection across rMED groups.

**Table 1:** Participant characteristics overall and across tertiles of Mediterranean diet adherence score (rMED) (n=3296)

	Means±SD or N (%)				P value
	Overall	Low rMED	Medium rMED	High rMED	
Age (years)	68.18±7.43	68.59±7.51	69.24±7.32	69.79±7.45	0.002
Men	1519 (46.1%)	457 (44.5%)	669 (47.7%)	393 (45.4%)	0.64
Women	1777 (53.9%)	570 (55.5%)	734 (52.3%)	473 (54.6%)	
White ethnicity	3222 (97.8%)	1009 (98.2%)	1372 (97.8%)	841 (97.1%)	0.26
Age completed education (years)	16.89±1.63	16.58±1.55	16.92±1.63	17.23±1.66	<0.001
Household income (£/week)	510.35±517.54	437.41±325.36	511.11±426.60	595.55±765.82	<0.001
Limiting long-standing illness	966 (29.3%)	346 (33.7%)	400 (28.5%)	220 (25.4%)	<0.001
Self-rated health (fair or poor)	590 (17.9%)	250 (24.3%)	227 (16.2%)	113 (13.0%)	<0.001
Current smoker	209 (6.3%)	111 (10.8%)	72 (5.1%)	26 (3.0%)	<0.001
Physically active	2380 (72.2%)	664 (64.7%)	1036 (73.8%)	680 (78.5%)	<0.001
Mean energy intake (kcal/day)	2014.28±571.75	2027.24±599.9	2032.17±564.1	1970.12±547.3	0.029
rMED score (range 0–18)	5.81±2.55	2.89±1.13	5.94±0.80	9.07±1.21	<0.001
CASP-12 (range 0–36)					
2018–2019 (baseline)	27.63±5.45	26.59±5.92	27.83±5.24	28.53±5.00	<0.001
Summer 2020	26.61±5.86	25.73±6.28	26.68±5.73	27.49±5.44	<0.001
CES-D score (range 0–8)	1.08±1.57	1.26±1.71	1.03±1.53	0.93±1.45	<0.001
Experience of COVID-19 infection by summer 2020	218 (6.6%)	66 (6.4%)	87 (6.2%)	65 (7.5%)	0.37

CES-D, Center for Epidemiologic Studies Depression scale; rMED, relative Mediterranean Diet Index.

Cross-sectionally, greater adherence to the Mediterranean diet was associated with higher levels of psychological well-being (table 2). The strength of the relationship was reduced from β=0.164 (SE: 0.017, p<0.001) in model 1 to β=0.054 (SE: 0.014, p<0.001) in model 3 (representing the change in CASP-12 score for a one-point increase in rMED score), suggesting that a proportion of the association was explained by health status, lifestyle and sociodemographic covariates. Despite the decrease in effect size, the relationship remained significant. The change of coefficient from model 2 to 3 was minor, indicating that depression was not a major confounder of the association, and that positive well-being has an independent association with diet quality, regardless of depressive symptomatology. Despite being inversely correlated one with the other (β=−0.364 (0.015), p<0.001), these two constructs are not reflecting the same dimensions of mental health. The main results were replicated in the larger sample of 3953 participants, where the regression coefficient in model 3 was 0.070 (p<0.001, see online supplemental table S2).

**Table 2:** Cross-sectional associations of Mediterranean diet (rMED score) with psychological well-being (CASP-12 score) ELSA wave 9 (2018/19) (n = 3296)

	Model 1		Model 2		Model 3	
	Beta (SE)	P value	Beta (SE)	P value	Beta (SE)	P value
rMED score	0.164 (0.017)	<0.001	0.060 (0.015)	<0.001	0.054 (0.014)	<0.001
Age	−0.111 (0.17)	<0.001	−0.022 (0.015)	0.14	−0.043 (0.014)	0.002
Sex*	0.008 (0.018)	0.63	0.009 (0.015)	0.11	0.062 (0.014)	<0.001
Ethnicity†	−0.023 (0.017)	0.18	−0.012 (0.015)	0.44	−0.004 (0.014)	0.78
Energy intake	0.040 (0.018)	0.027	−0.007 (0.015)	0.65	−0.012 (0.014)	0.39
Income‡			0.130 (0.016)	<0.001	0.116 (0.015)	<0.001
Education			−0.004 (0.016)	0.79	−0.017 (0.015)	0.25
Limiting long-standing illness§			−0.133 (0.017)	<0.001	−0.092 (0.016)	<0.001
Self-rated health¶			−0.373 (0.018)	<0.001	−0.275 (0.017)	<0.001
Smoking status**			−0.036 (0.015)	0.017	−0.029 (0.014)	0.038
Physical activity††			0.056 (0.016)	<0.001	0.025 (0.015)	0.084
CES-D depression					−0.364 (0.015)	<0.001

* Reference category: male.

† White European.

‡ Income in deciles.

§ No limiting long-standing illness.

¶ Good self-rated health.

** Non-smoker.

†† No moderate/vigorous physical activity.

CES-D, Center for Epidemiologic Studies Depression scale; ELSA, English Longitudinal Study of Ageing; rMED, relative Mediterranean Diet Index.

Psychological well-being deteriorated on average between 2018/2019 and the early months of the COVID-19 pandemic in the complete ELSA cohort,^14^ and this pattern was replicated in the present subsample (p<0.001, see table 1). Baseline CASP-12 ratings were strong predictors of well-being during the pandemic (β=0.705, p<0.001), and experience of COVID-19 infection was also related to lower well-being (β=−0.057, p<0.001). Despite the average decrease in CASP-12, a higher rMED score in 2018/2019 was associated with less deterioration in CASP-12 during the pandemic in all three models (table 3). The sensitivity analysis showed a similar longitudinal association in the larger sample (β=0.028, p=0.018, see online supplemental table S3). Additionally, the association with well-being remained strong when depression ratings obtained during the pandemic replaced baseline depression as a covariate (β=0.030, p=0.007 in fully adjusted model).

**Table 3:** Longitudinal associations of Mediterranean diet (rMED score) in 2018–2019 with psychological well-being during the COVID-19 pandemic (CASP-12 score in summer 2020) (n= 3023)

	Model 1		Model 2		Model 3	
	Beta (SE)	P value	Beta (SE)	P value	Beta (SE)	P value
rMED score	0.027 (0.013)	0.034	0.026 (0.013)	0.046	0.026 (0.013)	0.042
Age	−0.023 (0.13)	0.069	−0.022 (0.013)	0.096	−0.026 (0.013)	0.045
Sex*	−0.054 (0.013)	<0.001	−0.055 (0.013)	<0.001	−0.046 (0.013)	<0.001
Ethnicity†	0.024 (0.013)	0.064	0.025 (0.013)	0.053	0.027 (0.013)	0.035
Energy intake	0.010 (0.013)	0.43	0.007 (0.013)	0.62	0.005 (0.013)	0.71
Wave 9 CASP	0.705 (0.013)	<0.001	0.705 (0.014)	<0.001	0.640 (0.016)	<0.001
COVID-19 infection‡	−0.057 (0.013)	<0.001	−0.055 (0.013)	<0.001	−0.050 (0.013)	<0.001
Income§			0.003 (0.014)	0.81	0.004 (0.014)	0.76
Education			−0.036 (0.014)	0.008	−0.040 (0.014)	0.004
Limiting long-standing illness¶			−0.049 (0.015)	<0.001	−0.045 (0.015)	0.002
Self-rated health**			−0.050 (0.015)	<0.001	−0.041 (0.015)	0.006
Smoking status††			−0.011 (0.013)	0.41	0.011 (0.013)	0.40
Physical activity‡‡			0.009 (0.014)	0.51	0.006 (0.014)	0.68
CES-D depression					−0.070 (0.015)	<0.001

* Reference category: male.

† White European.

‡ No COVID-19 infection.

§ Income in deciles.

¶ No limiting long-standing illness.

** Good self-rated health.

†† Non-smoker.

‡‡ No moderate/vigorous physical activity.

CES-D, Center for Epidemiologic Studies Depression scale; rMED, relative Mediterranean Diet Index.

## Discussion

Our results indicate that greater adherence to a Mediterranean diet is associated with higher levels of positive psychological well-being in a large sample of men and women aged 50 years and older. The association was independent of sociodemographic factors, measures of health and health-related behaviours and also of depressive symptoms. This is consistent with research showing that positive well-being and mental ill-health have distinct associations with behavioural and health outcomes.^6^ Additionally, we found that greater adherence to the Mediterranean diet measured in 2018/2019 was related to a smaller decrement in positive psychological well-being in the early months of the COVID-19 pandemic in this population cohort.

The nutrition module was administered online which was a departure from the face-to-face interviewing carried in other parts of ELSA. The response rate was 49.8%, indicating that a substantial number of survey participants were unable or unwilling to complete the module. Whether this was because the module was online or because it concerned nutrition is difficult to know. Other studies have shown that more than 80% of men and women in ELSA indicated that they regularly used the internet, but some may have been reluctant to complete quite a complicated assessment.^33^ Interestingly, people who did not complete the nutrition module were not older than those who did, but they were less well educated and had lower incomes on average. They also reported more illness, poorer self-rated health, less favourable health behaviour and lower psychological well-being. These differences indicate that findings should be interpreted cautiously.

The analyses took account of a wide range of factors that potentially confound the association. As expected on the basis of previous research, psychological well-being was greater among people with higher incomes, better health and more physical activity, while being lower among smokers.^34^,^36^ Adherence to the Mediterranean diet is also often correlated with higher education and income, better health, higher levels of physical activity and lower smoking rates.^37 38^ However, the relationship of psychological well-being with Mediterranean diet remained significant when these factors were taken into account. The association was also independent of total energy intake, suggesting that the findings are not a result of overall undernutrition in older people who experience low psychological well-being.

Research relating the Mediterranean diet with positive psychological well-being has been limited. Several studies have shown links between different measures of psychological well-being and fruit and vegetable consumption,^9 39 40^ but these have not involved comprehensive dietary assessments. Trudel-Fitzgerald *et al*^41^ computed the Alternate Healthy Diet Index in the Nurses’ Health Study, in combination with measures of smoking, physical activity and alcohol consumption, and found that women in that study with higher happiness levels were more likely to report sustained healthy lifestyles over 10–22 years of follow-up. The Mediterranean diet is a distinct composite dietary index that appears relevant to positive psychological well-being as well as depression. A cross-sectional nationwide survey in Spain found that higher adherence to a Mediterranean diet was associated with lower negative affect and positively with overall evaluative well-being.^42^ More recently, an intervention study in adults aged 40–55 years with a modified Mediterranean-DASH Intervention for Neurodegenerative Delay (MIND) diet) showed improvement in positive affect (but no change regarding negative affect) and health-related quality of life in the intervention arm after 12 weeks compared with a control group who had dietary recommendations.^43^

The cross-sectional results can be affected by reverse causality: it is both possible that individuals with a higher psychological well-being select healthier diets or that the Mediterranean diet promotes positive well-being. More insight can be gained from the longitudinal analysis, since the COVID-19 pandemic challenged the well-being of the population.^13 14^ Experiences during the pandemic make it possible to test the protective association of diet on well-being under conditions of widespread social, health and economic disruption. We found that although psychological well-being declined during the early months of the pandemic, the reduction was smaller among individuals who reported greater adherence to the Mediterranean diet. The association was independent of depressive symptoms and direct experience of COVID-19 infection. Causal conclusions cannot be drawn from this observational study, but the results are consistent with a protective effect of the Mediterranean diet.

The biological processes underlying these associations are uncertain. Various elements of the Mediterranean diet, in particular dietary fibre, omega-3 fatty acids, micronutrients such as magnesium or polyphenols and the lower intake of pro-inflammatory food such as processed meat or dairy, are known to modulate stress responses, inflammation, oxidative stress, neuroplasticity, mitochondrial function, the gut microbiome and gut health.^1 44^ There is also some evidence that adherence to a Mediterranean diet during the COVID-19 pandemic was associated with greater physical activity, which may itself promote psychological well-being.^45^

Unfortunately, constraints on the length of assessments during the pandemic in ELSA prohibited detailed dietary assessments, so we do not know whether participants continued their prepandemic dietary practices. Other limitations to the study include the selective completion of the nutrition module detailed earlier, and the assessment of diet being based on just two 24-hour recalls. This method is valuable for large population studies but introduces measurement error.^46^ Psychological well-being was captured on only one occasion early in the pandemic in June/July 2020, and there is evidence for mental health being worse in April and May of that year.^47^ The study sample was predominantly of white ethnic background, so results may not generalise to other groups.

Nevertheless, we could confirm the cross-sectional association with longitudinal analyses in a very specific context of overall deterioration of the population’s well-being, therefore providing novel and robust evidence of the association between adherence to a Mediterranean diet and greater well-being in English older adults. This emphasises the importance of policies and recommendations targeting a healthy lifestyle, in particular a healthy balanced diet rich in plant-based food in the older population, not only to maintain physical health but also mental health and overall well-being.

## Supplementary material

10.1136/bmjopen-2025-109599 online supplemental file 1

## Data Availability

Data are available in a public, open access repository.
