# Supplementary material for: Adherence to the Mediterranean diet and psychological wellbeing before and during the COVID-19 pandemic: a prospective analysis of the English Longitudinal Study of Ageing
Source: BMJ Open. 2026 Jun 16;16(6):e109599. doi: 10.1136/bmjopen-2025-109599 (PMC13289082; doi:10.1136/bmjopen-2025-109599)
Supplement: online supplemental file 1 [file bmjopen-16-6-s001.docx]

**Adherence to the Mediterranean diet and psychological wellbeing before and during the Covid-19 pandemic: A prospective analysis of the English Longitudinal Study of Ageing**

**Supplementary Material**

**Table S1** Comparison of participants in ELSA Wave 9 who did and did not complete the nutrition module

**Table S2** Cross-sectional associations of Mediterranean diet (rMED score) with psychological wellbeing

ELSA Wave 9 (2018/19) (excluding income) (n = 3,953)

**Table S3** Longitudinal associations of Mediterranean diet (rMED score) with psychological wellbeing during the Covid-19 pandemic, excluding income (n= 3,644)

**Supplementary table S1 Characteristics of participant with and without nutrition data**

|  | Nutrition data  collected  (n = 4,254) | Nutrition data  Not collected  (n = 4,303) | *p* |
| --- | --- | --- | --- |
| Age (years)  Missing n | 68.31 ± 7.74  0 | 68.52 ± 12.82  0 | 0.37 |
| Men  Women  Missing n | 1946 (45.7%)  2301 (54.3%)  0 | 1864 (43.3%)  2439 (56.7%)  0 | 0.025 |
| White ethnicity  Missing n | 4143 (97.4%)  0 | 4000 (93.1%)  6 | <0.001 |
| Age completed education (yrs)  Missing n | 16.81 ± 1.75  0 | 16.23 ± 1.75  0 | <0.001 |
| Household income (£/week)  Missing n | 507.23 ± 510.17  701 | 386.73 ± 329.34  751 | <0.001 |
| Limiting long-standing illness  Missing n | 1257 (29.6%)  4 | 1719 (40.0%)  6 | <0.001 |
| Self-rated health (fair or poor)  Missing n | 804 (18.9%)  2 | 1257 (33.2%)  512 | <0.001 |
| Current smoker  Missing n | 281 (6.6%)  0 | 538 (12.5%)  2 | <0.001 |
| Physically active  Missing n | 3064 (72.0%)  0 | 2403 (55.9%)  1 | <0.001 |
| CASP-12 (range 0 -36)  Missing n | 27.65 ± 5.51  242 | 25.43 ± 6.30  1,348 | <0.001 |
| CES-D score (range 0-8)  Missing n | 1.09 ± 1.59  20 | 1.68 ± 2.00  500 | <0.001 |
|  |  |  |  |

**Supplementary Table S2 Cross-sectional associations of Mediterranean diet (rMED score) with psychological wellbeing**

**ELSA Wave 9 (2018/19) (excluding income) (n = 3,953)**

|  | Model 1 | | | Model 2 | | | Model 3 | |
| --- | --- | --- | --- | --- | --- | --- | --- | --- |
|  | Beta (SE) | p | Beta (SE) | | p | Beta (SE) | | p |
| rMED score | 0.172 (0.016) | <0.001 | 0.079 (0.014) | | <0.001 | 0.070 (0.013) | | <0.001 |
| Age | -0.105 (0.16) | <0.001 | -0.020 (0.014) | | 0.15 | -0.040 (0.013) | | 0.003 |
| Sex^a^ | 0.023 (0.016) | 0.15 | 0.004 (0.014) | | 0.78 | 0.052 (0.013) | | <0.001 |
| Ethnicity^b^ | -0.032 (0.016) | 0.039 | -0.026 (0.014) | | 0.063 | -0.018 (0.013) | | 0.16 |
| Energy intake | 0.031 (0.016) | 0.055 | -0.013 (0.014) | | 0.38 | -0.010 (0.013) | | 0.44 |
| Education |  |  | -0.031 (0.014) | | 0.033 | -0.016 (0.013) | | 0.23 |
| Limiting long-standing illness^c^ |  |  | -0.135 (0.016) | | <0.001 | -0.094 (0.015) | | <0.001 |
| Self-rated health^d^ |  |  | -0.378 (0.017) | | <0.001 | -0.274 (0.016) | | <0.001 |
| Smoking status^e^ |  |  | -0.028 (0.014) | | 0.046 | -0.023 (0.013) | | 0.069 |
| Physical activity^f^ |  |  | 0.065 (0.015) | | <0.001 | 0.037 (0.014) | | 0.007 |
| CES-D depression |  |  |  | |  | -0.367 (0.014) | | <0.001 |

Reference category: ^a^Male; ^b^White European; ^c^No limiting long-standing illness; ^d^Good self-rated health; ^e^Non-smoker; ^f^No moderate/vigorous physical activity

**Supplementary Table 3 Longitudinal associations of Mediterranean diet (rMED score) with psychological wellbeing**

**during the Covid-19 pandemic, excluding income (n= 3,644)**

|  | Model 1 | | | Model 2 | | | Model 3 | |
| --- | --- | --- | --- | --- | --- | --- | --- | --- |
|  | Beta (SE) | p | Beta (SE) | | p | Beta (SE) | | p |
| rMED score | 0.030 (0.012) | 0.010 | 0.028 (0.012) | | 0.020 | 0.028 (0.012) | | 0.018 |
| Age | -0.025 (0.12) | 0.032 | -0.023 (0.012) | | 0.055 | -0.027 (0.012) | | 0.024 |
| Sex^a^ | -0.060 (0.012) | <0.001 | -0.062 (0.012) | | <0.001 | -0.054 (0.012) | | <0.001 |
| Ethnicity^b^ | 0.034 (0.012) | 0.003 | 0.035 (0.012) | | 0.003 | 0.036 (0.012) | | 0.002 |
| Energy intake | 0.006 (0.012) | 0.61 | 0.002 (0.012) | | 0.88 | 0.002 (0.012) | | 0.89 |
| Wave 9 CASP | 0.703 (0.012) | <0.001 | 0.703 (0.013) | | <0.001 | 0.636 (0.014) | | <0.001 |
| Covid-19 infection^c^ | -0.048 (0.012) | <0.001 | -0.045 (0.013) | | <0.001 | -0.040 (0.012) | | <0.001 |
| Education |  |  | -0.029 (0.012) | | 0.015 | -0.032 (0.012) | | 0.008 |
| Limiting long-standing illness^d^ |  |  | -0.048 (0.014) | | <0.001 | -0.045 (0.013) | | <0.001 |
| Self-rated health^e^ |  |  | -0.053 (0.014) | | <0.001 | -0.043 (0.014) | | 0.002 |
| Smoking status^f^ |  |  | -0.009 (0.012) | | 0.45 | -0.009 (0.012) | | 0.43 |
| Physical activity^g^ |  |  | 0.014 (0.012) | | 0.25 | 0.012 (0.012) | | 0.35 |
| CES-D depression |  |  |  | |  | -0.073 (0.014) | | <0.001 |

Reference category: ^a^Male; ^b^White European; ^c^No Covid-19 infection; ^d^No limiting long-standing illness; ^e^Good self-rated health; ^f^Non-smoker; ^g^No moderate/vigorous physical activity
