# Supplementary material for: Adherence to the Mediterranean diet and psychological wellbeing before and during the COVID-19 pandemic: a prospective analysis of the English Longitudinal Study of Ageing
Source: BMJ Open. 2026 Jun 16;16(6):e109599. doi: 10.1136/bmjopen-2025-109599 (PMC13289082; doi:10.1136/bmjopen-2025-109599)
Supplement: Supplementary file 2 [file bmjopen-2025-109599.reviewer_comments.pdf]

PEER REVIEW HISTORY

BMJ Open publishes all reviews undertaken for accepted manuscripts. Reviewers are asked to complete a checklist review form and are provided with free text boxes to elaborate on their assessment. These free text comments are reproduced below.

ARTICLE DETAILS

Title (Provisional)

Adherence to the Mediterranean diet and psychological wellbeing before and during the Covid-19 pandemic: A prospective analysis of the English Longitudinal Study of Ageing

Authors

Steptoe, Andrew; Shand, Alanna Jo; Lassale, Camille

VERSION 1 - REVIEW

**Reviewer**	**1**
**Name**	**Goyena, Eva A**
**Affiliation**	**Food and Nutrition Research Institute, FNRI-DOST**
**Date**	**03-Feb-2026**
**COI**	**None**

This is a good study examining the association of healthy diets and wellbeing using validated dietary and wellbeing measures, along with the appropriate multivariable modelling analysis.

To further strengthen the manuscript, minor revisions are suggested: 1) to include how missing data were handled in the analysis; 2) to add statement on how potential selection bias of older participants 50-90 years old during the Covid-19 survey was avoided since only those with internet were able to participate during the pandemic; 3) to include justification why participants were grouped into tertiles of intake using relative Mediterranean Diet Index (rMEd) indicated in page 5 and 7, for clarity.

In additional, also indicate whether dietary intake using 24 Hr Food Recalls was average, and how within person variability was addressed during analysis..

**Reviewer**	**2**
**Name**	**Kelson, Zoe**
**Affiliation**	**University of Exeter, Mathematics**
**Date**	**14-Apr-2026**

COI **None**

This study investigated whether adherence to a Mediterranean diet is associated with positive wellbeing among older adults in England, and determined whether an emotionally protective relationship remained during the Covid-19 pandemic.

Reviewer comments:

"Data were drawn from the English Longitudinal Study of Ageing (ELSA), involving 3,296 adults aged 50 and older" [Abstract]

and

"The English Longitudinal Study of Ageing (ELSA) is a population survey of a representative sample of men and women ranging in age from 50 to over 90 years, living in the community in England (Steptoe et al, 2013). Data are collected at two year intervals, with new respondents being periodically added to maintain the full age range...

online nutritional assessment was included in Wave 9 (2018/19) and was completed both by ELSA core participants and by non-core individuals, such as younger partners or older people in the household who were not part of the representative sample...

Measures of psychological wellbeing and all covariates bar income were completed by 3,953 of the remaining participants. Income in ELSA is only assessed in the core sample, reducing the sample size for cross-sectional analyses to 3,296. The primary analyses were carried out on the core sample, but the analyses of the full sample (3,953) are included in supplemental material..."

and

"Of the participants with a complete nutrition assessment and covariates at wave 9, measures of psychological wellbeing during the pandemic were available from 3,023 individuals (91.6%)."

Can the authors please further assess and comment on representativeness of the analysed cohorts?

Can missing data and loss to follow-up be considered to be missing at random?

"For alcohol, participants falling in the recommended range of “moderate drinking” (range: 5–25 g/d ethanol for women and 10–50 g/d for men) were attributed a score of 2, 0 otherwise"

Is this per day?

To clarify, alcohol below or above these ranges are scored the same?

Given food components are scored in tertiles (i.e. relative to the rest of the cohort), can alcohol also be considered in this way (e.g. in tertiles)?

"The study sample was divided into tertiles of rMED scores (allowing for ties) for descriptive purposes. Differences between rMED groups were analysed by analysis of variance for continuous and χ 2 tests for categorical variables respectively."

Can the authors please confirm if and how they assessed normality for the application of parametric ANOVA tests?

"Multivariable linear regression models examined crosssectional and longitudinal associations between diet and wellbeing. " [Abstract]

and

"The cross-sectional association of Mediterranean diet and psychological wellbeing was analysed using multivariable linear regression, with results presented as standardised regression coefficients (β) with standard errors and p values. Three regression models were tested, with CASP-12 scores as the continuous outcome. Model 1 included age, gender, ethnicity and total energy intake (kcal/day) along with the main exposure, the rMED score (also continuous). Model 2 additionally adjusted for demographic, health and behaviour variables, namely income, education, limiting long-standing illness, low selfrated health, smoking and physical activity. Model 3 tested whether associations of diet quality and positive wellbeing were present after negative mood states were taken into account, so the CES-D was added to the analysis.

The longitudinal relationship between Mediterranean diet in 2018/2019 and psychological wellbeing during the Covid-19 pandemic was analysed by using CASP-12 measures taken in June/July 2020 as the outcome variable. In addition to the variables included in the crosssectional analyses, Model 1 included baseline CASP-12 scores and the experience of Covid-19 infection. Models 2 and 3 added the same variables as before"

Appropriate modelling methods have been applied by the authors.

"Supplementary analyses tested the cross-sectional and longitudinal associations in the full sample, after excluding income from the models. We also evaluated whether the longitudinal relationship remained robust if CES-D depression scores obtained during the pandemic were substituted for baseline depression. "

Insightful additional analyses have been explored by the authors.

The Results are reported accurately and concisely.

"Table 1 Participant characteristics (n = 3,296) "

In addition to tabulating the demographics of the full cohort, can core and longitudinal cohort demographics please also be tabulated (and shown in the supplementary material)?

"Unfortunately, constraints on the length of assessments during the pandemic in ELSA prohibited detailed dietary assessments, so we do not know whether participants continued their prepandemic dietary practices. Other limitations to the study include the assessment of diet being based on just two 24h recalls. This method is valuable for large population

studies, but introduces measurement error (Delerue Matos et al, 2021). Psychological wellbeing was captured on only one occasion early in the pandemic in June/July 2020, and there is evidence for mental health being worse in April and May of that year (Fancourt et al, 2021). The study sample was predominantly of White ethnic background, so results may not generalise to other groups."

The authors provide a Discussion on the study limitations.

Thanks for providing a copy of the STROBE checklist.

VERSION 1 - AUTHOR RESPONSE

Reviewer: 1
Dr. Eva A Goyena, Food and Nutrition Research Institute
Comments to the Author:
This is a good study examining the association of healthy diets and wellbeing using validated dietary and wellbeing measures, along with the appropriate multivariable modelling analysis.

To further strengthen the manuscript, minor revisions are suggested: 1) to include how missing data were handled in the analysis; 2) to add statement on how potential selection bias of older participants 50-90 years old during the Covid-19 survey was avoided since only those with internet were able to participate during the pandemic; 3) to include justification why participants were grouped into tertiles of intake using relative Mediterranean Diet Index (rMEd) indicated in page 5 and 7, for clarity.

1. A complete case analysis was carried out because missing data on covariates was less than 0.01% (page 8).

2. We have added a section concerning internet assessments on page 5-6, focusing on the nutrition module which was also online. We have added a table to the supplementary material (Supplement 1) comparing ELSA participants who did and did not complete the nutrition module. It can be seen from this that individuals who completed this online module were more advantaged socioeconomically and in terms of health than those who did not provide data. We emphasise the relevance of this to the generalisability of findings both in the 'strengths and limitations' section and in the Discussion (page 11).

3. The grouping of the sample into tertiles was purely for descriptive purposes, and we have added a phrase to emphasis to page 8. This grouping was not used in the statistical analyses relating Mediterranean diet with psychological wellbeing, but to give readers a general idea of the associations between dietary intake and covariates.

In additional, also indicate whether dietary intake using 24 Hr Food Recalls was average, and how within person variability was addressed during analysis..

We averaged data from the two dietary assessment days, and this is now indicated on page 6 of the revised manuscript.

Reviewer: 2
Prof. Zoe Kelson, University of Exeter
Comments to the Author:
This study investigated whether adherence to a Mediterranean diet is associated with positive wellbeing among older adults in England, and determined whether an emotionally protective relationship remained during the Covid-19 pandemic.

Reviewer comments:

"Data were drawn from the English Longitudinal Study of Ageing (ELSA), involving 3,296 adults aged 50 and older" [Abstract]
and
"The English Longitudinal Study of Ageing (ELSA) is a population survey of a representative sample of men and women ranging in age from 50 to over 90 years, living in the community in England (Steptoe et al, 2013). Data are collected at two year intervals, with new respondents being periodically added to maintain the full age range...
online nutritional assessment was included in Wave 9 (2018/19) and was completed both by ELSA core participants and by non-core individuals, such as younger partners or older people in the household who were not part of the representative sample...
Measures of psychological wellbeing and all covariates bar income were completed by 3,953 of the remaining participants. Income in ELSA is only assessed in the core sample, reducing the sample size for cross-sectional analyses to 3,296. The primary analyses were carried out on the core sample, but the analyses of the full sample (3,953) are included in supplemental material..."
and
"Of the participants with a complete nutrition assessment and covariates at wave 9, measures of psychological wellbeing during the pandemic were available from 3,023 individuals (91.6%)."
Can the authors please further assess and comment on representativeness of the analysed cohorts?
We have added a table (Supplement 1) comparing individuals who completed the online nutrition assessment with those who did not. It can be seen from this that individuals who completed this online module were more advantaged socioeconomically and in terms of health than those who did not provide data. We emphasise the relevance of this to the generalisability of findings both in the ‘strengths and limitations’ section and in the Discussion These differences are described on page 5-6 and discussed on page 11.

Can missing data and loss to follow-up be considered to be missing at random?
Beyond what is noted in the previous comment regarding the selection of the sample, missing data on specific covariates can be considered to be missing at random. Loss to follow-up is likely linked to mental and physical health, which therefore might have incurred selection bias. However, the reasonably low percentage of participants lost to follow-up (7.8%) in our analysis minimizes this risk.

"For alcohol, participants falling in the recommended range of "moderate drinking" (range: 5–25 g/d ethanol for women and 10–50 g/d for men) were attributed a score of 2, 0 otherwise"
Is this per day?

Yes the unit if gram/day. This was alcohol consumed on the days of dietary assessment, collected as part of the dietary module. So this is the number of grams consumed on those days. This is now indicated on page 7.

To clarify, alcohol below or above these ranges are scored the same?

Yes, in the rMED categorisation, alcohol consumption above and below the specified range is scored the same way (assigned a score of 0), whereas intake falling within the range is assigned a score of 2.

Given food components are scored in tertiles (i.e. relative to the rest of the cohort), can alcohol also be considered in this way (e.g. in tertiles)?

We were following the categorisation in the original rMED score, which has been published, validated and used in many different settings. We did not think it appropriate to change their categorisation because that would reduce comparability with earlier studies.

"The study sample was divided into tertiles of rMED scores (allowing for ties) for descriptive purposes. Differences between rMED groups were analysed by analysis of variance for continuous and χ 2 tests for categorical variables respectively."
Can the authors please confirm if and how they assessed normality for the application of parametric ANOVA tests?

As noted above to answer to the first reviewer, this grouping was not used in the statistical analyses relating Mediterranean diet with psychological wellbeing, only for descriptive purposes to give readers a general idea of the associations between dietary intake and covariates.

"Multivariable linear regression models examined crosssectional and longitudinal

associations between diet and wellbeing. " [Abstract]
and
"The cross-sectional association of Mediterranean diet and psychological wellbeing was analysed using multivariable linear regression, with results presented as standardised regression coefficients (β) with standard errors and p values. Three regression models were tested, with CASP-12 scores as the continuous outcome. Model 1 included age, gender, ethnicity and total energy intake (kcal/day) along with the main exposure, the rMED score (also continuous). Model 2 additionally adjusted for demographic, health and behaviour variables, namely income, education, limiting long-standing illness, low self-rated health, smoking and physical activity. Model 3 tested whether associations of diet quality and positive wellbeing were present after negative mood states were taken into account, so the CES-D was added to the analysis.
The longitudinal relationship between Mediterranean diet in 2018/2019 and psychological wellbeing during the Covid-19 pandemic was analysed by using CASP-12 measures taken in June/July 2020 as the outcome variable. In addition to the variables included in the crosssectional analyses, Model 1 included baseline CASP-12 scores and the experience of Covid-19 infection. Models 2 and 3 added the same variables as before"
Appropriate modelling methods have been applied by the authors.
Thank you.

"Supplementary analyses tested the cross-sectional and longitudinal associations in the full sample, after excluding income from the models. We also evaluated whether the longitudinal relationship remained robust if CES-D depression scores obtained during the pandemic were substituted for baseline depression. "
Insightful additional analyses have been explored by the authors.

The Results are reported accurately and concisely.

"Table 1 Participant characteristics (n = 3,296) "
In addition to tabulating the demographics of the full cohort, can core and longitudinal cohort demographics please also be tabulated (and shown in the supplementary material)?

We have added Supplementary Table 1 to address this issue.

"Unfortunately, constraints on the length of assessments during the pandemic in ELSA prohibited detailed dietary assessments, so we do not know whether participants continued their prepandemic dietary practices. Other limitations to the study include the assessment of diet being based on just two 24h recalls. This method is valuable for large population studies, but introduces measurement error (Delerue Matos et al, 2021). Psychological wellbeing was captured on only one occasion early in the pandemic in June/July 2020, and there is evidence for mental health being worse in April and May of that year (Fancourt et al,

2021). The study sample was predominantly of White ethnic background, so results may not generalise to other groups."
The authors provide a Discussion on the study limitations.

Thanks for providing a copy of the STROBE checklist.

Reviewer: 1
If you have selected ‘Yes’ above, please provide details of any competing interests.: Not applicable

Reviewer: 2
If you have selected ‘Yes’ above, please provide details of any competing interests.: Not applicable
